# Development of roGFP2-derived redox probes for measurement of the glutathione redox potential in the cytosol of severely glutathione-deficient *rml1* seedlings

**DOI:** 10.3389/fpls.2013.00506

**Published:** 2013-12-16

**Authors:** Isabel Aller, Nicolas Rouhier, Andreas J. Meyer

**Affiliations:** ^1^INRES-Chemical Signalling, University of BonnBonn, Germany; ^2^Interactions Arbres Microorganismes, IFR 110 EFABA, Faculté des sciences, Université de Lorraine, UMR 1136 Université de Lorraine/INRAVandoeuvre lès-Nancy, France

**Keywords:** glutathione, glutathione redox potential, GRX1-roGFP2, *rml1*, redox imaging

## Abstract

Glutathione is important for detoxification, as a cofactor in biochemical reactions and as a thiol-redox buffer. The cytosolic glutathione buffer is normally highly reduced with glutathione redox potentials (*E*_*GSH*_) of more negative than −310 mV. Maintenance of such negative redox potential is achieved through continuous reduction of glutathione disulfide by glutathione reductase (GR). Deviations from steady state glutathione redox homeostasis have been discussed as a possible mean to alter the activity of redox-sensitive proteins through switching of critical thiol residues. To better understand such signaling mechanisms it is essential to be able to measure *E*_*GSH*_ over a wide range from highly negative redox potentials down to potentials found in mutants that show already severe phenotypes. With the advent of redox-sensitive GFPs (roGFPs), understanding the *in vivo* dynamics of the thiol-based redox buffer system became within reach. The original roGFP versions, roGFP1 and roGFP2, however, have midpoint potentials between −280 and −290 mV rendering them fully oxidized in the ER and almost fully reduced in the cytosol, plastids, mitochondria, and peroxisomes. To extend the range of suitable probes we have engineered a roGFP2 derivative, roGFP2-iL, with a midpoint potential of about −238 mV. This value is within the range of redox potentials reported for homologous roGFP1-iX probes, albeit with different excitation properties. To allow rapid and specific equilibration with the glutathione pool, fusion constructs with human glutaredoxin 1 (GRX1) were generated and characterized *in vitro*. GRX1-roGFP2-iL proved to be suitable for *in vivo* redox potential measurements and extends the range of *E*_*GSH*_ values that can be measured *in vivo* with roGFP2-based probes from about −320 mV for GRX1-roGFP2 down to about −210 mV for GRX1-roGFP2-iL. Using both probes in the cytosol of severely glutathione-deficient *rml1* seedlings revealed an *E*_*GSH*_ of about −260 mV in this mutant.

## Introduction

Thiol redox biochemistry is considered to play a fundamental role in cellular processes including signaling and cell fate decisions. The ability to dynamically and quantitatively measure such cellular processes *in vivo* is key to understand the underlying principles and the coordination of redox processes in the context of intact cells. Redox-sensitive GFP (roGFP) allows a direct read-out of the glutathione redox potential (*E*_*GSH*_) particularly in reducing compartments (Meyer and Dick, 2010). However, current variants of roGFPs are largely inadequate in mutants with very low glutathione levels, generally oxidizing conditions like in the ER, or oxidizing conditions triggered by pathological processes. These limitations demand the development of further roGFP variants with less negative midpoint potentials.

The tripeptide glutathione (γ-L-glutamyl-L-cysteinylglycine) constitutes the major low molecular weight thiol in most prokaryotic and virtually all eukaryotic organisms. Glutathione is synthesized in two sequential steps that are catalyzed by two enzymes, glutamate-cysteine ligase (GSH1) and glutathione synthase (GSH2). In plants, glutathione fulfills a broad range of essential functions including detoxification of heavy metals and xenobiotics and serving as an electron donating cofactor in biochemical reactions (Cobbett and Goldsbrough, 2002; Noctor et al., 2011). Moreover glutathione constitutes one of the most important redox buffer systems in the cell. The capacity to act as a redox buffer relies on the reversible convertibility of glutathione between the reduced form of glutathione (GSH) and the oxidized form glutathione disulfide (GSSG). While the glutathione pool in the cytosol, mitochondria, plastids, and peroxisomes is maintained in a highly reduced state by NADPH and glutathione reductase (GR) (Schwarzländer et al., 2008; Marty et al., 2009), the glutathione redox buffer in the endoplasmic reticulum (ER) is highly oxidized (Hwang et al., 1992; Brach et al., 2009). *E*_*GSH*_ depends on the absolute glutathione concentration and the ratio of [GSH]:[GSSG] (Meyer and Hell, 2005). The local *E*_*GSH*_ is assumed to affect the glutathionylation status of proteins which is an important means of regulating protein activity (Michelet et al., 2005; Noctor et al., 2011; Zaffagnini et al., 2012).

Conditions of environmental challenge are frequently considered to cause the generation of oxidants in the form of reactive oxygen species (ROS) (Apel and Hirt, 2004; Torres et al., 2006). Apart from potential toxic effects of ROS, the oxidants are increasingly recognized as vital messengers in cellular signaling (Finkel, 2011). Oxidant-dependent signaling may occur either directly by specific recognition of oxidants (Forman et al., 2010) or indirectly through detoxification of oxidants and resulting changes in the cellular redox buffer system providing an opportunity for readout of these changes. Stress-dependent alterations of *E*_*GSH*_ caused by a transient draw of electrons from the glutathione redox buffer for oxidant detoxification and coupling of *E*_*GSH*_ to target proteins thus may result in altered protein function and hence pronounced metabolic and developmental consequences for individual cells and the whole organism.

Depletion of total GSH also directly impacts on *E*_*GSH*_ and thus mutants with defects in GSH biosynthesis may cause constitutive activation or inactivation of respective signaling pathways. Indeed, Arabidopsis *rax1* mutants carrying a mutation in the GSH1 enzyme leading to only 20–50% of wild-type GSH are affected in stress signaling and have induced defense pathways (Ball et al., 2004). Another partially GSH-deficient mutant, *pad2*, is not capable of activating appropriate defense mechanisms against biotic stressors (Parisy et al., 2007; Schlaeppi et al., 2008). The most severe, yet viable, mutant affected in GSH1 is *rml1*, which contains only 2–10% of wild-type glutathione (Vernoux et al., 2000; Cairns et al., 2006). Homozygous *rml1* mutant seedlings show impaired root development, which is assumed to be caused by redox-dependent inhibition of cell cycle components (Vernoux et al., 2000).

Better understanding of redox-regulatory processes mediated by glutathione demands experimental approaches enabling quantitative monitoring of *E*_*GSH*_. The development of roGFPs enables ratiometric thiol redox imaging at subcellular level (Dooley et al., 2004; Hanson et al., 2004). The two excitation maxima of the parental GFP arise from the protonation state of the chromophore and excited state proton transfer that converts the neutral form of the chromophore into the green emitting anionic form (Brejc et al., 1997; Palm et al., 1997). Engineering of two surface-exposed cysteines into the GFP barrel on the two adjacent β-strands 7 and 10 in positions allowing reversible disulfide formation exploits a structure-dependent shift in the protonation status of the chromophore for ratiometric measurements (Dooley et al., 2004; Hanson et al., 2004). The degree of chromophore protonation is also dependent on the type of chromophore: while roGFP1 derived from the wild-type chromophore with the amino acid S65 in the chromophore displays a dominant excitation peak in the UV range, the roGFP2 variant derived from EGFP containing the S65T mutation has a main excitation peak at 490 nm (Hanson et al., 2004). The chromophore type also slightly affects the redox potential of the engineered disulfide with roGFP1 being slightly more negative than roGFP2. It has been shown that glutaredoxin (GRX) catalyzes the oxidation and reduction of roGFP in the presence of glutathione (Meyer et al., 2007; Gutscher et al., 2008). Fusion of GRX to roGFP overcomes kinetic limitations of the GRX/roGFP interaction and possible limitations caused by the absence of appropriate GRXs in some organisms or subcellular compartments (Gutscher et al., 2008; Meyer and Dick, 2010). In addition to kinetic properties, the thermodynamic properties of roGFP probes are similarly important for dynamic measurements and ideally redox sensors with redox potentials adapted to the desired measuring range should be selected. It has been shown that the redox potentials of roGFP variants, roGFP3 and roGFP4, which both contain the engineered disulfide C149/C202, are more negative than the redox potentials of their respective counterparts roGFP1 and roGFP2, which both contain the disulfide C147/C204 (Hanson et al., 2004). The redox potential can also be shifted to less negative values by engineering basic amino acids next to the disulfide-forming cysteines. As a consequence, such probes show an increased pI and hence a significantly enhanced response rate for reductive processes (Cannon and Remington, 2006). In the oxidizing ER lumen and under highly severe stress conditions, however, these probes would still be fully oxidized. To overcome this limitation, Lohman and Remington (2008) further engineered roGFP1 by replacing the chromophore-interacting H148 by serine and inserting an additional amino acid between the disulfide forming C147 and S148. Depending on the amino acid inserted behind C147 the respective members of the so-called roGFP1-iX family had less negative midpoint potentials between −229 and −246 mV (Lohman and Remington, 2008). However, even these less reducing probes appear almost fully oxidized in the ER at steady state of HeLa cells (Birk et al., 2013).

While under highly reducing conditions roGFP1 may have some advantages, roGFP2 has advantages in less reducing conditions as they occur in partially glutathione-deficient mutants (Meyer et al., 2007; Schwarzländer et al., 2008). Specifically, roGFP2 has a larger dynamic range than roGFP1 and it avoids potential illumination-dependent photoisomerization artifacts associated with roGFP1 (Schwarzländer et al., 2008). Because roGFP2 also is less reducing than roGFP1 we asked whether roGFP2-iX probes can be engineered and whether such probes would have an even less negative redox potential than their respective roGFP1-iX counterparts to enable redox imaging in compartments with a less negative *E*_*GSH*_. Inhibition of GSH biosynthesis in wild-type roots by BSO leads to severe oxidation in the cytosol (Meyer et al., 2007). Similarly, *E*_*GSH*_ in *rml1* roots has been shown to be far less negative than in wild-type plants (Meyer and Dick, 2010), but the exact value of cytosolic *E*_*GSH*_ in this mutant has not been determined because the redox potential is close to the edge or even beyond the usable measuring range of roGFP2. After detailed characterization the novel roGFP2-iL probe was thus used to determine *E*_*GSH*_ in the cytosol of *rml1* seedlings in order to define the lower limit of glutathione redox potentials under which viability can be maintained.

## Materials and methods

### Gene construction, protein expression and purification

roGFP2 (C48S/S65T/Q80R/F99S/S147C/Q204C, (Dooley et al., 2004; Hanson et al., 2004) was used as a template to generate roGFP2-iL (C48S/S65T/Q80R/F99S/S147CL/H148S/Q204C) in which a leucine was added after C147. For site-specific insertion of C147CL and substitution of H148S into the roGFP2 sequence the primers 5′-AACTACAACTGCCTGAGCAACGTCTATATCATGGCC-3′ and 5′-GCTCAGGCAGTTGTAGTTGTACTCCAGCTTGTG-3′ were used. N-terminal fusion of human GRX1 and roGFP2-iL was done by PCR using gene-specific primers 5′-TCAGGAGGAGTGAGCAAGGGCGA-3′ and 5′-TCGCCCTTGCTCACTCCTCCTGA-3′. Amplification of full-length product was done with the primer pair 5′-ACCATGATGGCTCAAGAGTTTGTGAA-3′ and 5′-TCTAGACTTGTACAGCTCGTCCATG-3′ generating GRX1-TS(GGSGG)_6_-roGFP2-iL (GRX1-roGFP2-iL). Both PCR products were cloned into pCAP^*s*^ vector (Roche, www.roche-applied-science.com) for sequence confirmation.

The sequence of free roGFP2-iL and GRX1-roGFP2-iL were cloned into the BamHI and NcoI restriction sites of the protein expression vector pQE-30 (Qiagen, www.qiagen.com) for the production of a recombinant protein containing an N-terminal His_6_-tag. The restriction sites were introduced by PCR using the primer pairs 5′-TGGATCCGCTCAAGAGTTTGTGAACTG-3′ and 5′-CTAAGCTTTTACTTGTACAGCTCGTCC-3′ for GRX1-roGFP2iL and 5′-AAGGATCCGTGAGCAAGGGCGAGGAGC-3′ and 5′-CTAAGCTTTTACTTGTACAGCTCGTCC-3′ for roGFP2-iL. Recombinant roGFP1 was produced as described earlier (Schwarzländer et al., 2008). pQE-30 plasmids for expression of roGFP1-iL and roGFP1-iE were kindly provided by Dr. J. Remington (Univ. Oregon) and Dr. C. Appenzeller-Herzog (Univ. Basel), respectively.

For stable expression in plants, a modified version of pBinAR vector (Höfgen and Willmitzer, 1990) containing UBQ10 promoter instead of 35S promoter was used. The coding sequence of GRX1-roGFP2iL was amplified by 5′-AGGTACCATGGCTCAAGAGTTTGTGAAC-3′ and 5′-TATGTCGACTTACTTGTACAGCTCGTCCAT-3′ to add KpnI and SalI restriction sites used for cloning.

### Isolation of recombinant proteins

After transformation of the *E. coli* strain Origami (DE3) (Novagen, www.merckmillipore.de), a pre-culture of 10 ml was grown over night at 37°C. Five mL of the pre-culture were added to 450 ml LB medium and grown at room temperature to an OD_600_ of 0.5–0.8. Expression of the different roGFP variants was induced by addition of isopropyl-β-D-thiogalactopyranoside (IPTG) to a final concentration of 1 mM. Protein expression was performed at room temperature for 24 h. The cells were harvested and resuspended in protein extraction buffer (50 mM Tris-HCl pH 8.0, 250 mM NaCl). The cells were sonicated for 10 min. Cell lysate was centrifuged and the soluble roGFP proteins purified via a Ni^2+^ loaded HiTrap™ Chelating HP Column (GE Healthcare, www.gelifesciences.com).

### Spectroscopy

Fluorescence excitation spectra were collected using a LS55 fluorescence spectrophotometer (Perkin Elmer Life Sciences, http://www.perkinelmer.com). Five hundred microliter protein solution was placed in a 1 ml quartz cuvette. Samples contained 0.2 μM protein in reaction buffer (100 mM K_2_HPO_4_/KH_2_PO_4_ pH 7.4, 1 mM EDTA) and 10 mM total dithiothreitol (DTT) for full reduction or 10 mM total H_2_O_2_ for full oxidation of the sensor, respectively. The spectra were collected from 350 to 520 nm with a bandwidth of 10 nm and a scan speed of 500 nm min^−1^. Fluorescence was detected at 540 nm.

The fluorescence quantum yield (QY) of roGFPs was determined by comparison to Rhodamine 6G (Sigma-Aldrich, www.sigmaaldrich.com) which has a QY of 0.9 when dissolved in water (Magde et al., 2002). Six different dilutions of the respective proteins with absorbances between 0.01 and 0.1 were prepared in aqueous solution. Both, standard and roGFP samples were excited at 488 nm with a bandwidth of 5 nm. Total emission was collected from 505 to 590 nm. Fluorescence quantum yields for roGFPs were calculated from the integrated fluorescence intensities of the spectra after correction for wavelength-dependent photomultiplier sensitivity.

### *In vitro* characterization of roGFP variants

*In vitro* characterization of roGFP2-iL and GRX1-roGFP2-iL fusions by ratiometric time-course measurements with isolated proteins was performed on a fluorescence plate reader (POLARstar Omega; BMG Labtech, www.bmglabtech.com) with filter-based excitation at 390 and 480 nm and detection of emitted light at 520 nm. Phosphate buffer (100 mM K_2_HPO_4_/KH_2_PO_4_, 1 mM EDTA, pH 7.4) with 1 μM of the respective roGFP according to the information given in the text were pipetted into the wells of a 96-well plate with a clear bottom (NUNC™ 96, www.thermoscientific.com). Reduced glutathione (in 100 mM K_2_HPO_4_/KH_2_PO_4_ buffer, pH 7.0) was automatically injected to the indicated final concentration using the built-in injectors. For maximum achievable reduction of the glutathione buffer, 0.1 μM recombinant *Arabidopsis thaliana* GR1 (*At*GR1) and 100 μM NADPH were added to each well. To allow comparable reduction kinetics, the proteins were pre-oxidized with 10 mM H_2_O_2_ for 30 min. The remaining H_2_O_2_ was removed by desalting spin columns according to the manufacturer's manual (Zeba™ Spin Desalting Columns, www.thermoscientific.com). H_2_O_2_ and DTT to a final concentration of 10 mM were separately used to define maximum oxidation and maximum reduction of the sensors. In the comparative reduction assays with free roGFP1, roGFP1-iL, roGFP2, and roGFP2iL, purified *At*GRX2 to a final concentration of 2 μM was included in the reaction mix.

### Determination of midpoint potentials of roGFP variants

Both roGFP2-iL and GRX-roGFP2-iL (1 μM final concentration) were allowed to equilibrate (2–3 h) with lipoic acid buffers [reduced form dihydrolipoic acid (DHLA); oxidized form lipoic acid (LA)] (Equation 1). DHLA/LA was used at a total concentration of 2.5 mM in degassed HEPES buffer (100 mM HEPES, 300 mM NaCl, 1 mM EDTA, pH 7.0). The appropriate concentrations of DHLA and LA to set distinct redox potentials were calculated from the Nernst equation based on the standard reduction potential of lipoic acid (*E*°′_*LA*_) of −290 mV at pH 7.0 (Lees and Whitesides, 1993).

(1) $${\text{roGFP}}_{\text{ox}}+\text{DHLA}\Leftrightarrow {\text{roGFP}}_{\text{red}}+\text{LA}$$

The redox potential for each of the two redox pairs is defined by the Nernst equation using the respective midpoint potential and ratios (*Q*) of oxidized to reduced roGFP and LA to DHLA, respectively (Equation 2).

(2) $$E=E^{{}^{\circ}\prime }-\frac{RT}{nF}\ln Q$$

In this equation, *R* is the gas constant (8.314 J K^−1^ mol^−1^), *T* is the temperature (298.15 K), *n* is the number of transferred electrons, and *F* is Faraday's constant (96,485 C mol^−1^). After redox equilibration of both redox couples it is possible to equate the two redox potentials (Equation 3):

(3) $$\begin{array}{l} E_{LA}=E_{LA}^{{}^{\circ}\prime }-\frac{0.0592V}{2}\log\frac{\left[\text{DHLA}\right]}{\left[\text{LA}\right]}=E_{roGFP}^{{}^{\circ}\prime }\\ \;-\frac{0.0592V}{2}\log\frac{\left[{\text{roGFP}}_{\text{red}}\right]}{\left[{\text{roGFP}}_{\text{ox}}\right]}=E_{roGFP} \end{array}$$

Because *E*°′_LA_ is known this leaves two unknowns, *E*°′_roGFP_ and [roGFP_red_]/[roGFP_ox_]. The redox potential of roGFP is dependent on the degree of oxidation (*OxD*_*roGFP*_) of the redox pair roGFP_red_/roGFP_ox_ according to Equations 4 and 5:

(4) $$OxD_{roGFP}=\frac{\left[{\text{roGFP}}_{\text{ox}}\right]}{\left[{\text{roGFP}}_{\text{ox}}]+[{\text{roGFP}}_{\text{red}}\right]}$$

(5) $$\frac{\left[{\text{roGFP}}_{\text{red}}\right]}{\left[{\text{roGFP}}_{\text{ox}}\right]}=\frac{1-OxD_{roGFP}}{OxD_{roGFP}}$$

Substitution of Equation 5 into Equation 3 allows transformation of Equation 3 into Equation 6:

(6) $$\begin{array}{l} E_{LA}=E_{LA}^{{}^{\circ}\prime }-\frac{0.0592V}{2}\log\frac{\left[\text{DHLA}\right]}{\left[\text{LA}\right]}=E_{roGFP}^{{}^{\circ}\prime }\\ \;-\frac{0.0592V}{2}\log\frac{1-OxD_{roGFP}}{OxD_{roGFP}}=E_{roGFP} \end{array}$$

*OxD*_*roGFP*_ can be measured *in vitro* by monitoring the fluorescence emission intensities (*I*) at 520 nm for excitation at 390 and 480 nm. After equilibration in an environment of a defined redox potential, the respective values were used to determine *OxD*_*roGFP*_ according to Equation 7:

(7) $$OxD_{roGFP}=\frac{R-R_{red}}{\left(\frac{I480_{\text{ox}}}{I480_{\text{red}}}\right)(R_{ox}-R)+(R-R_{red})}$$

Here, *R* denotes the ratio of the fluorescence intensities measured at 390 and 480 nm. *R*_*red*_ and *R*_*ox*_ represent the fluorescence ratios of fully reduced and fully oxidized roGFP, respectively. The raw values of *I* were always corrected by subtracting the respective blank values.

For determination of the midpoint potentials *E*°′ of roGFP variants *OxD*_*roGFP*_ or, alternatively, the degree of reduction *ReD*_*roGFP*_ = 1 − *OxD*_*roGFP*_ was plotted against the calculated redox potentials of the respective lipoic acid redox buffers and all data points were fitted to a sigmoidal dose-response curve using GraphPadPrism5 (GraphPad Software, www.graphpad.com). The titration of each protein was carried out three times with 4 technical replicates. Titration of GRX1-roGFP2 and roGFP2 was done with DTT, which has a more negative reduction potential than lipoic acid (*E*°′_DTT_ = −323 mV, Shaked et al., 1980).

### Plant material and growth conditions

Heterozygous Arabidopsis *rml1* mutants were selected by genotyping for the mutant allele and exploiting the fact that the point mutation (Vernoux et al., 2000) generates a new ApoI restriction site. After transformation of heterozygous plants with the respective roGFP constructs transformed plants were first screened for uniform fluorescence on a stereomicroscope equipped with fluorescence optics and a GFP filter. In a subsequent molecular screen heterozygous *rml1* seedlings were selected for further propagation. Seeds from transgenic plants expressing either GRX1-roGFP2 of GRX1-roGFP2-iL were surface sterilized with 70% ethanol twice and resuspended in sterile deionized water. Seeds were plated on nutrient medium [5 mM KNO_3_, 2.5 mM KH_2_PO_4_, 2 mM MgSO_4_, 2 mM Ca(NO_3_)_2_, 10 μM Fe-EDTA, 0.1% (v/v) micronutrient mix (Somerville and Ogren, 1982), pH 5.8 solidified with 0.8% phytagel]. Plants were kept at 4°C for 1 day before placing them in vertical orientation in a growth chamber with a diurnal cycle of 16 h light at 22°C and 8 h dark at 18°C. The light intensity was 75 μmol photons m^−2^ s^−1^. Plants were grown for 3 days until the characteristic dwarf phenotype of *rml1* became visible.

### CLSM imaging

Pre-selected *rml1* seedlings were mounted on a slide in a drop of water and immediately transferred to a Zeiss confocal microscope LSM780 (Carl Zeiss Microscopy, www.zeiss.de/mikro). Images were collected with a 40× lens (Zeiss Objective C-Apochromat 40×/1.2 W Corr M27) in multi-track mode with line switching between 488 nm excitation and 405 nm excitation and taking an average of four readings in case of GRX1-roGFP2 and two readings for GRX1-roGFP2-iL, respectively. The roGFP fluorescence was collected with a 505–530 nm emission band-pass filter. Autofluorescence excited at 405 nm was collected from 430 to 480 nm and values were used to subtract autofluorescence bleeding into the roGFP channel as described previously (Schwarzländer et al., 2008; Samalova et al., 2013).

### Ratiometric image analysis

Images were imported into a custom written MatLab (The MathWorks, www.mathworks.de) analysis suite (M.D. Fricker, Dept. Plant Sciences, Oxford). The ratio analysis was performed on a pixel-by-pixel basis as *I*405/*I*488 following spatial averaging in (*x*,*y*) using a 3 × 3 kernel. Correction of *I*405 for autofluorescence bleeding into the 405 nm channel and subtraction of background signals for each channel was performed. The average background signal was typically measured from the vacuole of one of the cells. For pseudocolor display, the ratio was coded on a spectral color scale ranging from blue (fully reduced) to red (fully oxidized), with limits set by the *in situ* calibration. The calibration was done by incubation of *rml1* seedlings expressing GRX1-roGFP2 or GRX1-roGFP2-iL in 10 mM DTT and 25 mM H_2_O_2_, respectively, to drive the roGFPs to their fully reduced and fully oxidized forms. *OxD* of GRX1-roGFP2 and GRX1-roGFP2-iL expressed in the cytosol of *rml1* mutants was calculated according to Equation 7 albeit with wavelengths 405 nm and 488 nm used for excitation of roGFPs *in vivo*.

## Results

### Development and characterization of roGFP2-iL

**Figure 1:**
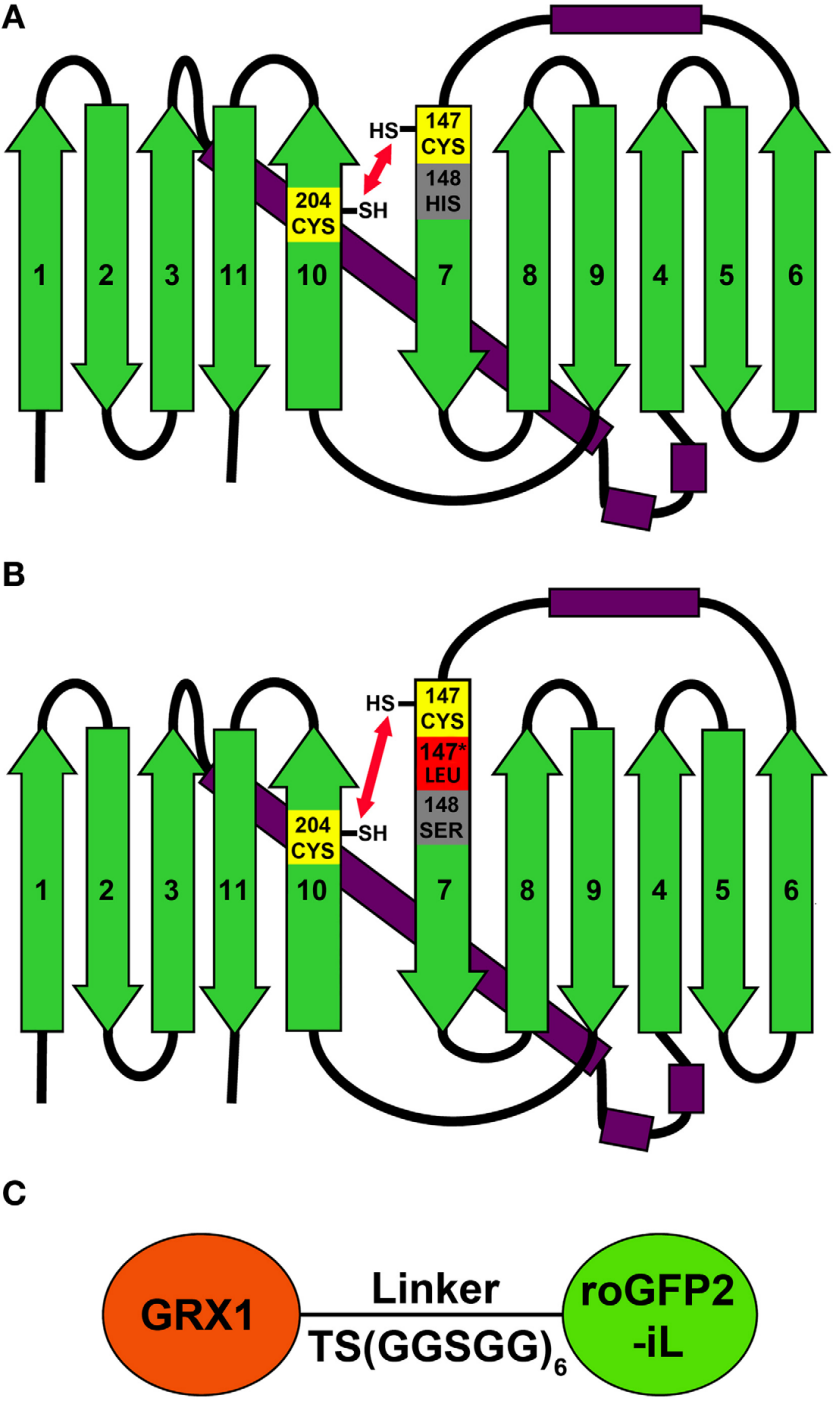
**2D models of roGFP2 and roGFP2-iL illustrate the relative position of disulfide forming Cys147 and Cys204. (A)** Cys147 and Cys204 are located on strands 7 and 10, respectively, and are positioned in close proximity so that they can facilitate disulfide formation. **(B)** The introduction of a leucine next to Cys147 (Leu147^*^) and the substitution of His148 by serine increase the relative distance between Cys147 and Cys204 that causes alterations in the thermodynamic stability of the disulfide. **(C)** Schematic representation of the GRX1-roGFP2-iL fusion protein. GRX1 was fused to the N-terminus of roGFP2-iL by a TS(GGSGG)_6_ linker.

**Figure 2:**
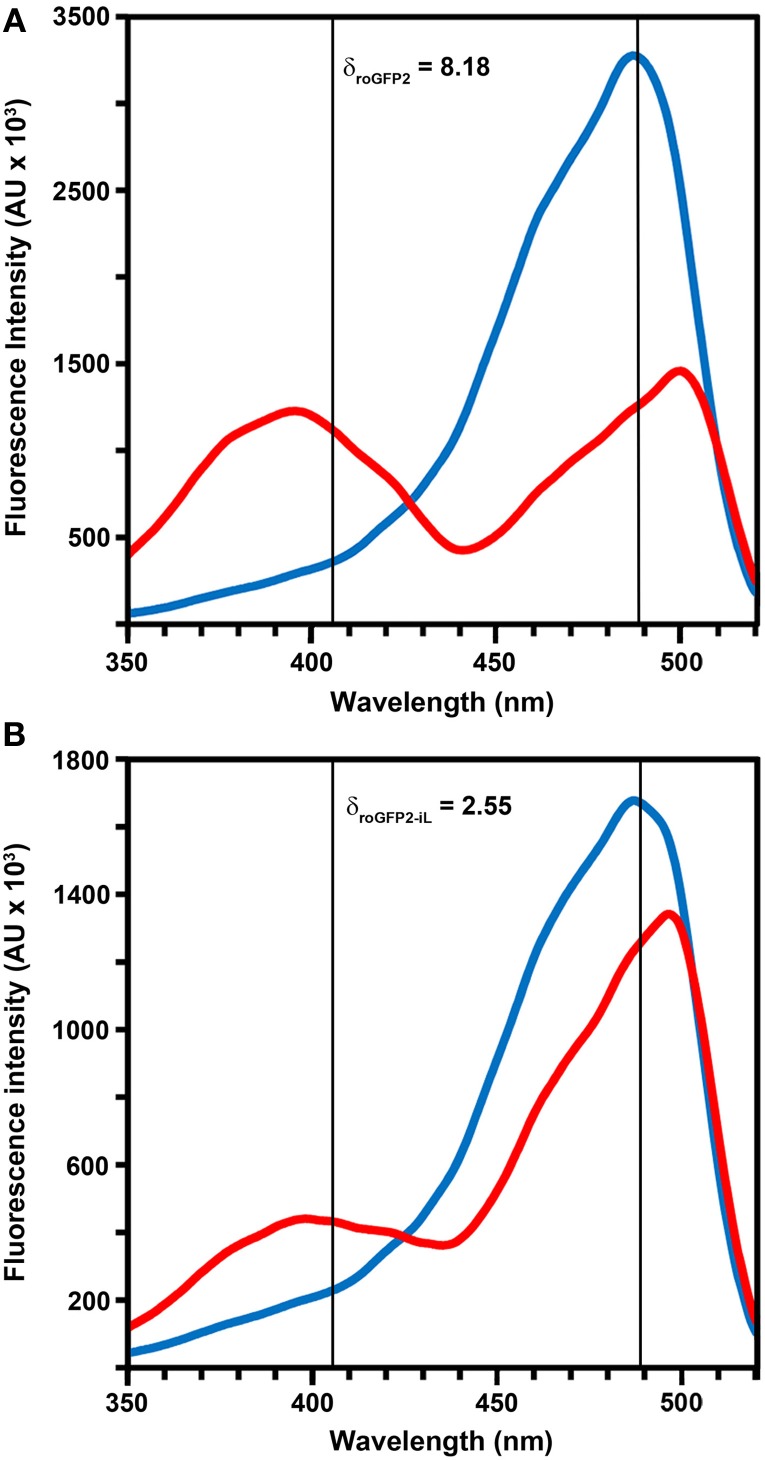
**Excitation properties of roGFP2 and roGFP2-iL.** Excitation spectra of roGFP2 **(A)** and roGFP2-iL **(B)** in fully oxidized (red curve) and fully reduced (blue curve) state. Emission was monitored at 540 nm. The maximum dynamic ranges (δ) were calculated from the 405/488 nm excitation ratios for fully reduced and fully oxidized probes. Both excitation wavelengths are indicated by vertical lines.

To investigate whether roGFP2 can be modified to generate less reducing probe variants, the roGFP2 sequence was further engineered by introducing site-specific mutations thus generating roGFP2-iL (Figures 1A,B). Subsequently, roGFP2-iL was fused behind human GRX1 to generate GRX1-roGFP2-iL (Figure 1C) to ensure specificity of the novel probe for *E*_*GSH*_ similar to other roGFP variants used before (Gutscher et al., 2008; Meyer and Dick, 2010; Albrecht et al., 2013; Birk et al., 2013). IPTG-induction of *E. coli* cultures transformed with roGFP2-iL and GRX1-roGFP2-iL already resulted in bright green cultures suggesting that the introduced mutations did not abolish the fluorescence (not shown). Comparison of roGFP2-iL and roGFP1-iE with the standard Rhodamine 6G resulted in a QY of 0.6 for roGFP2-iL and a QY of 0.4 for roGFP1-iE. This initial observation was further confirmed through side-by-side fluorescence scans of recombinant roGFP2 and roGFP2-iL in buffers containing either 10 mM DTT for full reduction or 10 mM H_2_O_2_ for full oxidation of the probes, respectively (Figure 2). As previously described (Dooley et al., 2004; Hanson et al., 2004), formation of the disulfide upon oxidation of roGFP2 favors the protonated, neutral form of the chromophore causing an increase in fluorescence at 395 nm while the 488 nm peak is decreased (red curve). Conversely, full reduction of roGFP2 leads to a decrease in excitation at 395 nm while the peak at 488 nm is increased (blue curve; Figure 2A).

**Figure 3:**
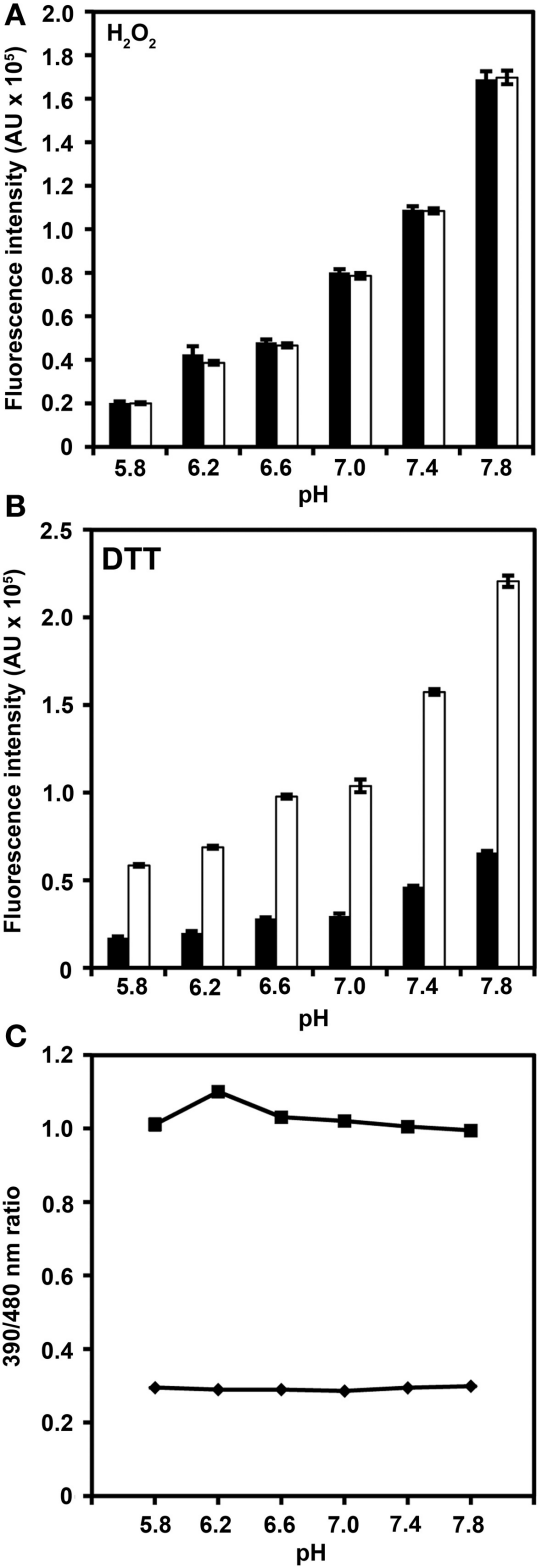
**pH-dependence of roGFP2-iL fluorescence.** Recombinant roGFP2-iL was incubated in potassium-phosphate buffer at different pH values. The changes in roGFP2-iL fluorescence intensity of the 390 nm (black) and 480 nm channel (white) in buffers of different pH values under full oxidation with 10 mM H_2_O_2_ **(A)** and full reduction by 10 mM DTT **(B)** is shown. Increasing pH values beyond pH 7.0 equally increase fluorescence intensity in both channels thus the 390/480 nm ratio remains unaffected. Conversely, decreasing pH below pH 7.0 has the opposite effect for fully oxidized and fully reduced roGFP2-iL. Similar pH-dependent effects have been reported for wild-type GFP (Patterson et al., 1997). **(C)** The excitation ratios (390/480 nm) in 10 mM H_2_O_2_ (♦) and in 10 mM DTT (■) of roGFP2-iL are plotted against the respective pH solution. For several data points, standard error is smaller than data marker (*n* = 3 technical replicates).

Modification of roGFP2 through the C147CL insertion and H148S exchange does alter the general spectral properties but does not abolish the dual excitation behavior in roGFP2-iL (Figure 2B). Full oxidation of the sensor causes an increase of fluorescence at 395 nm and a corresponding decrease in 488 nm excitation while full reduction of roGFP2-iL leads to decreased fluorescence excitation at 395 nm and an increase at 488 nm. A clear isosbestic point at 425 nm separates the two peaks further indicating two equilibrating molecular species (Figure 2B). Thus, all spectral properties of roGFP2 are fully retained in the newly-generated roGFP2-iL. In contrast to roGFP2, however, the maximum change in fluorescence intensity between fully oxidized and fully reduced sensor states was significantly lower in roGFP2-iL. The dynamic range (δ) for the maximum change of the fluorescence ratio between the fully oxidized and fully reduced state of the chromophore was calculated from the fluorescence excitation at the wavelengths used for ratiometric imaging (i.e., 405 and 488 nm). While roGFP2 has a δ_405/488_ of 8.18 the δ_405/488_ for roGFP2-iL was found to be 2.55 (Figure 2; Table 1). Further equilibration of roGFP2-iL in redox buffers with varying pH revealed that the fluorescence ratio of roGFP2-iL is pH-insensitive in the physiological range (Figure 3).

**Table 1:** **Mutations, redox potentials, and dynamic range of GFP derived redox probes**.

**Probe**	**Amino acid changes**	***E*°′**	**δ^a^**
roGFP2	C48S/S65T/Q80R/F99S/S147C/Q204C	−280 mV (consensus)	11.55 (390/480) 8.18 (405/488)
roGFP2-iL	C48S/S65T/Q80R/F99S/S147CL/H148S/Q204C	−240 mV (DHLA/LA)	3.1 (390/480) 2.55 (405/488)
roGFP1^b^^,^^c^	C48S/S147C/Q204C	−291 mV (consensus)	6.1 (400/475)^d^ 2.58 (405/488)^e^
roGFP1-iL^d^	C48S/Q80R/S147CL/H148S/Q204C	−229 mV (DHLA/LA)^f^	7.2 (400/475)^d^
roGFP1-iE^d^	C48S/Q80R/S147CE/H148S/Q204C	−236 mV (DHLA/LA)^f^	4.5 (400/475)^d^

a Dynamic range determined with the excitation wavelengths given in parenthesis. 405 nm and 488 nm are the excitation wavelengths used for confocal imaging.

b Hanson et al., 2004.

c Dooley et al., 2004.

d Lohman and Remington, 2008.

e Schwarzländer et al., 2008.

f Dihydrolipoic acid (DHLA, reduced form), Lipoic acid (LA, oxidized form).

**Figure 4:**
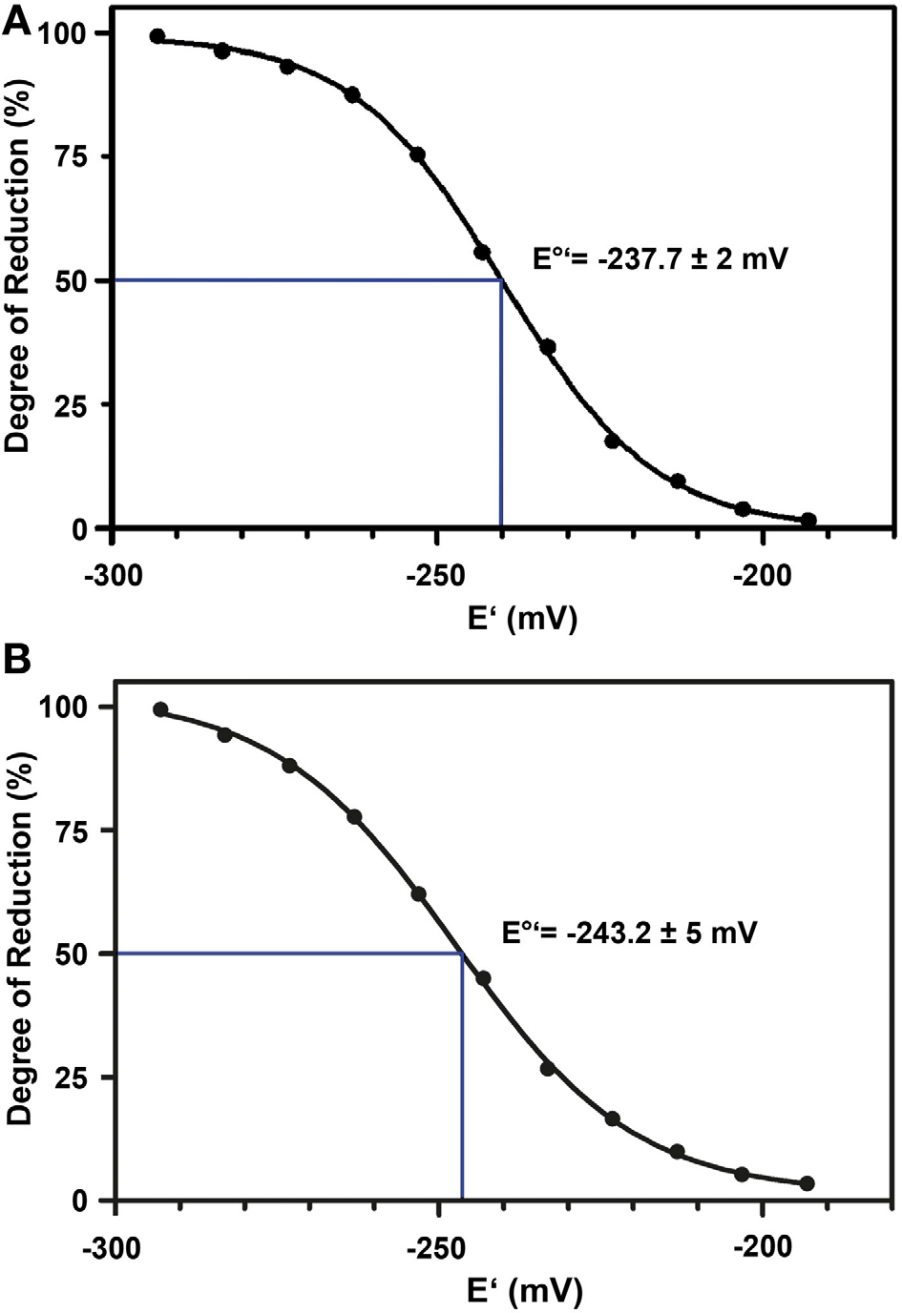
**Redox titration of roGFP2-iL and GRX1-roGFP2-iL.** The degree of reduction of roGFP2-iL **(A)** and GRX1-roGFP2-iL **(B)** was determined from the excitation ratios plotted against the potential of the ambient redox buffer. Representative measurements of at least 3 independent redox titrations (with 4 replicates each) for both probes are shown.

**Figure 5:**
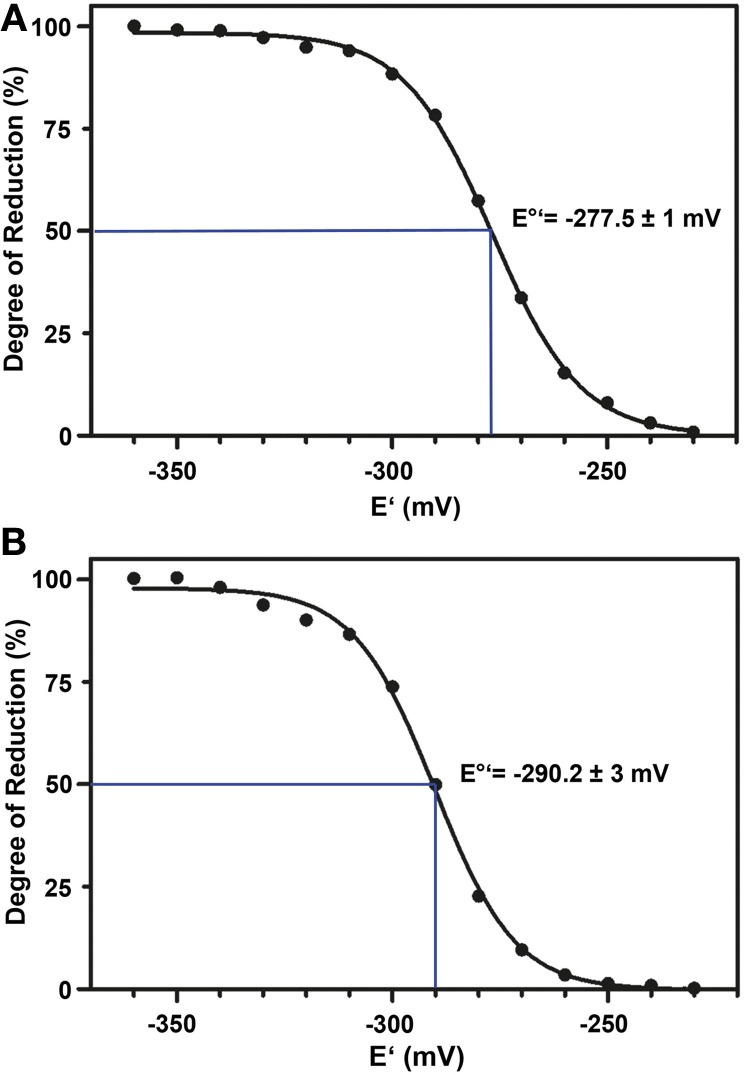
**Redox titration of roGFP2 and GRX1-roGFP2.** The degree of reduction of roGFP2 **(A)** and GRX1-roGFP2 **(B)** was determined from the excitation ratios plotted against the potential of the ambient redox buffer. Representative measurements of at least 3 independent redox titrations (with 4 replicates each) for both probes are shown.

The standard redox potential (*E*°′) strongly depends on the thermodynamic stability between C147 and C204 that is influenced by the nature of the surrounding amino acid residues. It has been described for roGFP1-iL that the insertion C147CL and the substitution H148S lower the thermodynamic stability of the inter-strand disulfide C147–C204 resulting in a less negative *E*°′ of the sensor (Lohman and Remington, 2008). To test whether this effect on thermodynamic stability is retained in roGFP2-iL, *E*°′_*roGFP2-iL*_ was determined through titration with LA/DHLA. Plotting the degree of reduction of the respective sensor variants against the redox potential of the ambient LA/DHLA buffer allowed to deduce *E*°′ values of −237.7 ± 2 mV for roGFP2-iL (Figure 4A) and 243.2 ± 5 mV for GRX1-roGFP2-iL (Figure 4B). The difference in *E*°′ of roGFP2-iL and GRX1-roGFP2-iL suggests that N-terminal fusion of GRX1 influences the thermodynamic properties of the C147–C204 disulfide. To test this hypothesis, the midpoint potential of roGFP2 and GRX1-roGFP2 was determined side-by-side by titration against DTT redox buffers. As depicted in Figure 5, free roGFP2 shows an *E*°′ of −277.5 ± 1 mV (Figure 5A) while *E*°′ of GRX1-roGFP2 is shifted to −290.2 ± 3 mV (Figure 5B).

**Figure 6:**
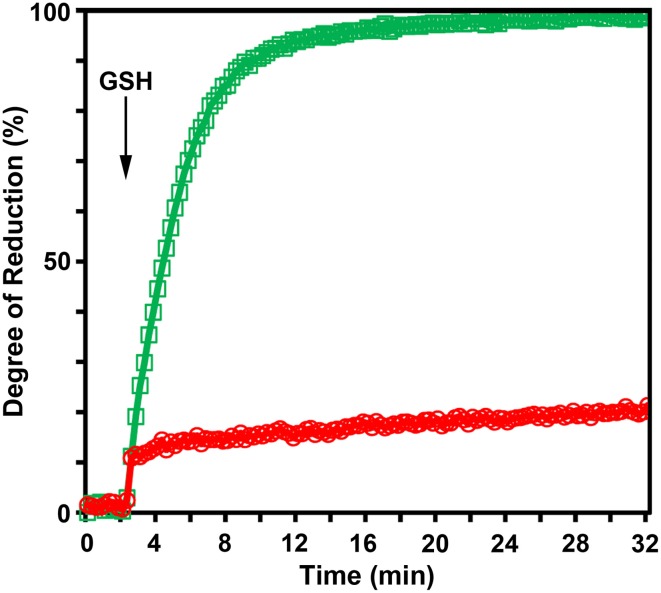
**The N-terminally fused glutaredoxin catalyzes the transfer of electrons from glutathione to roGFP2-iL.** The excitation ratio 390/480 nm with emission at 520 nm was followed over time. Fully reduced glutathione was added to a solution of oxidized roGFP2-iL (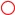) or GRX1-roGFP2-iL (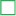), respectively 2.5 min after the start of the measurement. Change of reduction was recorded over time after injection of 2 mM reduced glutathione. The reaction buffer contained 100 μM NADPH and glutathione reductase at pH 7.4 to maintain full reduction of glutathione over the measured time (*n* = 4).

To test whether the reduction kinetics of roGFP2-iL is affected by GRX, roGFP2-iL was fused to the C-terminus of *Hs*GRX1 and the catalytic efficiency for the reduction of the resulting fusion protein by GSH was compared to free roGFP2-iL in *in vitro* experiments (Figure 6). In the absence of GRX, pre-oxidized roGFP2-iL responds only slowly to injection of 2 mM GSH while the GRX1-roGFP2-iL fusion protein shows a dramatically increased reduction rate.

**Figure 7:**
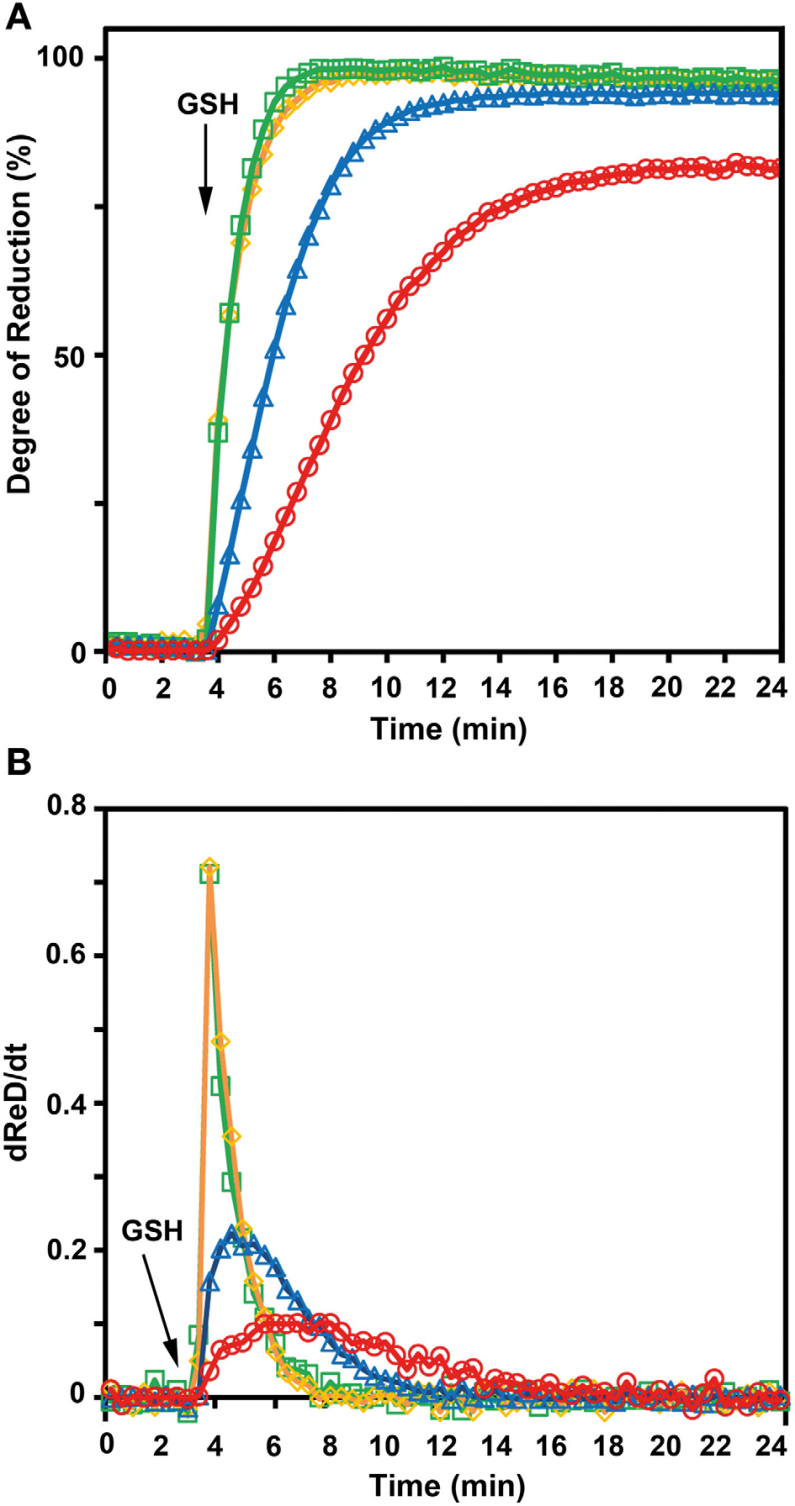
**GSH-dependent reduction of roGFP variants.** The kinetics for the reduction of roGFP by reduced glutathione strongly depends on redox potential of the disulfide forming cysteines of the respective roGFP sensors. **(A)** The excitation ratio 390/480 nm with emission at 520 nm was followed over time. Fully reduced glutathione (final concentration 5 mM) was added to a solution of pre-oxidized roGFP1-iL (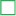), roGFP2-iL (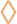), roGFP2 (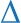), and roGFP1 (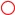) 3.75 min after start of the measurement. The reaction buffer contained 2 μM GRX, 100 μM NADPH, and glutathione reductase at pH 7.4 to maintain maximum reduction of glutathione over the measured time course (*n* = 4). **(B)** *dRed/dt* further illustrates the rapid change in reduction of roGFP1-iL (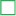) and roGFP2-iL (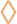) compared to roGFP2 (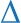) and roGFP1 (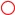).

To further confirm the less negative *E*°′ of roGFP2-iL, the sensor was used side-by-side with other roGFP variants in reduction experiments with GSH at physiological concentrations as reducing agent. Since not all compared roGFPs were available as fusion proteins with GRX1, recombinant free GRX was added to the reaction mix. Pre-oxidized roGFP1, roGFP2, roGFP1-iL and roGFP2-iL were all reduced in the presence of GSH which was injected into the roGFP solutions to a final concentration of 5 mM. Subsequently, the reduction kinetics of the sensors were followed over time (Figure 7A). Addition of GSH resulted in complete reduction of roGFP1-iL and roGFP2-iL within 3 min while the reduction of roGFP2 was clearly delayed by several minutes and reached only 95% within 21 min. The reduction of the most negative probe roGFP1 was even slower and reached only 81% within 21 min. The rapid change in the degree of reduction of sensors with significantly less negative *E*°′ values than *E*°′ values of roGFP2 and roGFP1 (Table 1) can be further illustrated by the first derivative *dReD/dt* of the degree of reduction. *dRed/dt* of the four different probes plotted against time after injection of GSH shows the most rapid change in the degree of reduction for roGFP1-iL and roGFP2-iL while the values for roGFP2 and roGFP1 are much lower (Figure 7B).

### Measurement of the cytosolic *E*_*GSH*_ in severely glutathione-deficient *rml1* mutants

**Figure 8:**
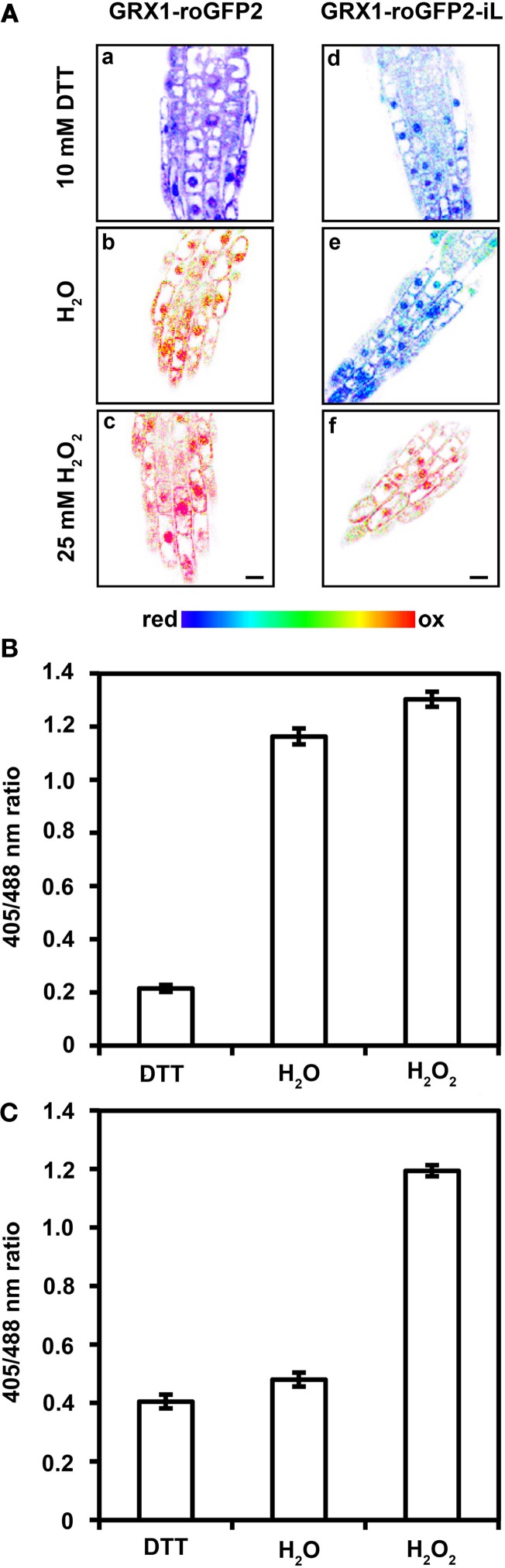
**The GRX1-roGFP2-iL probe expressed in the cytosol of homozygous *rml1* mutants is almost completely reduced while GRX1-roGFP2 is highly oxidized. (A)** Ratiometric images **(a–f)** show the redox state of GRX1-roGFP2 and GRX-roGFP2-iL in the cytosol of root epidermal cells of *rml1* mutants. Treatment with 10 mM DTT results in full reduction of GRX1-roGFP2 **(a)** and GRX-roGFP2-iL **(d)**, while under resting conditions GRX1-roGFP2 is highly oxidized **(b)** whereas GRX1-roGFP2-iL is in a reduced state **(e)**. Treatment with 25 mM H_2_O_2_ results in complete oxidation of GRX1-roGFP2 **(c)** and GRX-roGFP2-iL **(f)**. Scale bar 10 μm. (**B,C**) Fluorescence ratio of cytosolic GRX1-roGFP2 **(B)** and GRX1-roGFP2-iL **(C)** show complete reduction and oxidation of GRX-roGFP2 and GRX1-roGFP2-iL after treatment with 10 mM DTT and 25 mM H_2_O_2_, respectively. Under resting conditions in H_2_O, GRX1-roGFP2 is highly oxidized while GRX1-roGFP2-iL is highly reduced. Values are calculated as the mean ± SD (*n* = 5 − 10).

Conventional roGFP1 and roGFP2 are highly reduced when expressed in the cytosol of non-stressed cells (Meyer et al., 2007; Schwarzländer et al., 2008). Therefore, roGFP-iX probes with a less negative midpoint potential would obviously be expected to be also fully reduced, particularly when fused GRX1 ensures specific equilibration with the local *E*_*GSH*_. Severe stress situations and pathological conditions, however, can lead to pronounced oxidation in the cytosol and thus probes with a less negative midpoint potential may be advantageous for redox imaging in the cytosol to fully resolve the dynamic changes in *E*_*GSH*_. Severe depletion of GSH in the Arabidopsis mutant *rml1* has been reported to result in growth inhibition and it has been speculated that this effect may be caused by severe oxidation of cytosolic *E*_*GSH*_ and redox-dependent inhibition of the cell cycle (Vernoux et al., 2000). Indeed, roGFP2 has been shown to be largely oxidized in the cytosol of *rml1* seedlings (Meyer and Dick, 2010). To further investigate the effect of severe GSH depletion on the cytosolic *E*_*GSH*_, both probes, GRX1-roGFP2 and GRX1-roGFP2-iL were expressed in the cytosol of *rml1* and used to measure *E*_*GSH*_ in root epidermal cells. As expected, the conventional GRX1-roGFP2 was largely oxidized (Figures 8A,B). Incubation of *rml1* seedlings with 25 mM H_2_O_2_ resulted only in minor additional increase in the fluorescence ratio of GRX1-roGFP2 while incubation of seedlings in 10 mM DTT caused a pronounced drop in fluorescence ratio. Expression of GRX1-roGFP2-iL in the cytosol of *rml1* mutants, on the other hand, resulted in a largely reduced probe (Figures 8A,C). Full reduction of the probe through incubation of seedlings with 10 mM DTT caused only a small drop in the detected fluorescence ratio. In contrast, incubation with 25 mM H_2_O_2_ resulted in a pronounced increase in the 405/488 nm fluorescence ratio. The dynamic range of the probe in the cytosol calculated from the respective *min* and *max* ratios was about 3-fold (Figure 8C), which is similar to the predictions from the spectral analysis of recombinant roGFP2-iL (Figure 2).

**Figure 9:**
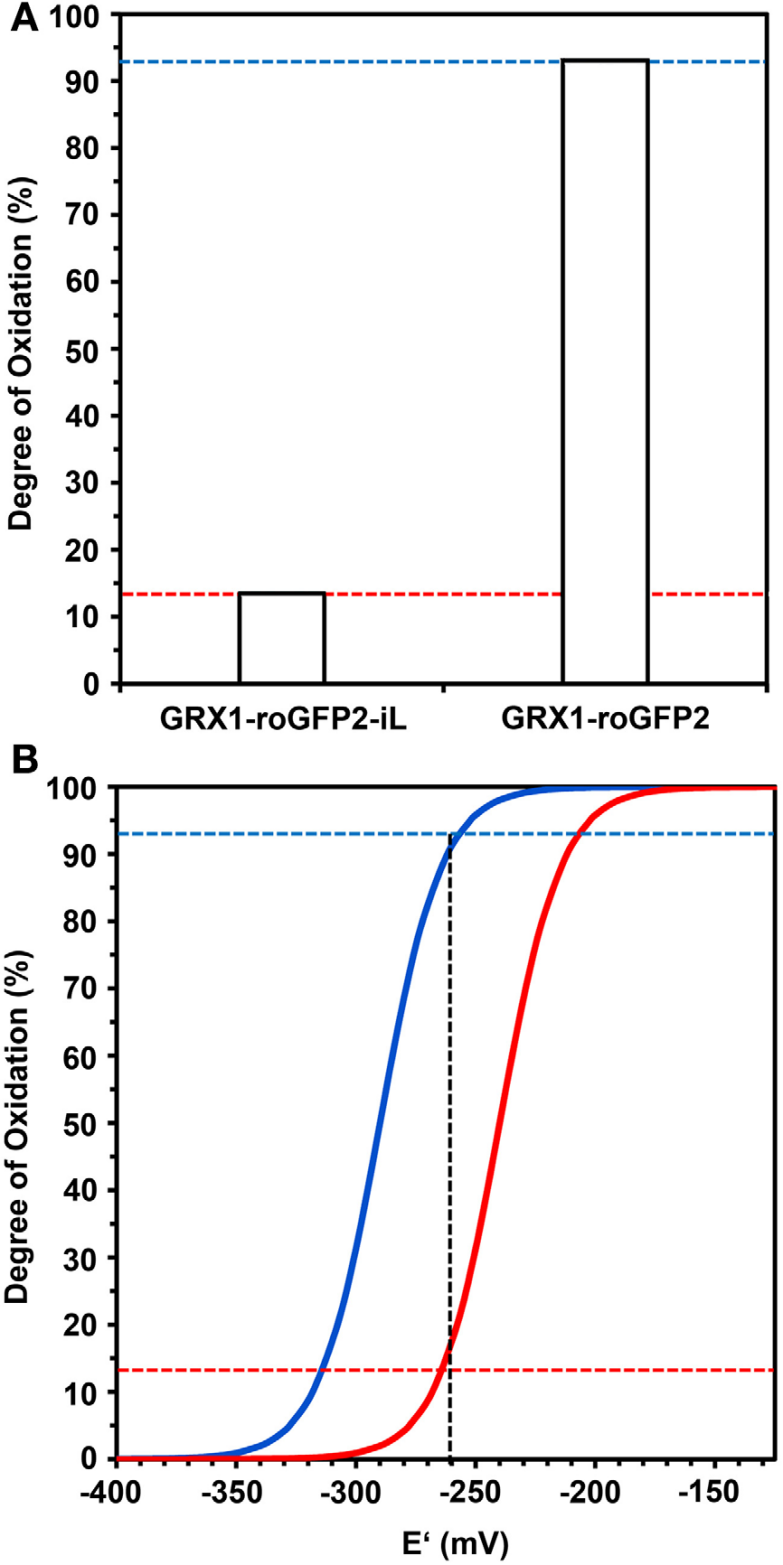
**Deduction of the redox potential of the roGFP probes from the Degree of Oxidation (*OxD*) in the cytosol of *rml1* seedlings. (A)** GRX1-roGFP2-iL and GRX1-roGFP2 expressed in the cytosol of *rml1* plants as calculated according to equations given in Materials and Methods from the mean fluorescence data presented in Figure 7. While GRX1-roGFP2 is highly oxidized only a small fraction of GRX1-roGFP2-iL is in the oxidized state. **(B)** Titration curves drawn for GRX1-roGFP2 (blue) and GRX1-roGFP2-iL (red) were calculated from the Nernst-Equation using the midpoint potentials determined in Figures 3, 4. Dotted horizontal lines refer to *OxD* of GRX1-roGFP2 and GRX1-roGFP2-iL from *rml1* measurements shown in panel **(A)**. The vertical dotted line indicates the interception points for GRX1-roGFP2 (blue) and GRX-roGFP2-iL (red) which suggests an *E*_*GSH*_ of about −260 mV in the cytosol of *rml1*.

Calculation of the degree of oxidation (*OxD*) of both probes from the ratiometric imaging data presented in Figure 8 showed a low *OxD* of only 14% for GRX1-roGFP2-iL and a corresponding high *OxD* of 93% for GRX1-roGFP2 (Figure 9A). The measured *OxD* values for both probes can be compared to titration curves for GRX1-roGFP2 (blue) and GRX1-roGFP2-iL (red) that were calculated from the Nernst-Equation using *E*°′ values determined earlier (Figure 9B). The interception points between the titration curves and the *OxD* for GRX1-roGFP2 and GRX1-roGFP2-iL, respectively, suggest an *E*_*GSH*_ in the cytosol of *rml1* of about −260 mV.

## Discussion

The glutathione redox potential in life cells varies depending on the compartment and on environmental conditions imposing stress situations for the plant. While cytosol, peroxisomes, mitochondria, and plastids in Arabidopsis wild-type plants maintain a highly reduced glutathione buffer with redox potentials of less than −310 mV, the ER is far less reducing with *E*_*GSH*_ values of less than −240 mV (Meyer et al., 2007; Schwarzländer et al., 2008). In nominally reducing compartments, severe stress can trigger physiological conditions frequently described as oxidative stress. Better understanding of the underlying molecular processes leading to gradual oxidation and also the downstream processes involved in signal transduction cascades enabling the plant to adapt to a stress situation requires the ability to measure individual components of the cellular redox buffer system in a dynamic way. The roGFPs, and particularly GRX1-roGFP2 fusion proteins, have been shown to sense the local *E*_*GSH*_ with high specificity due to selective mediation of thiol/disulfide exchange reaction between glutathione and roGFP by GRXs (Meyer et al., 2007; Gutscher et al., 2008). The originally introduced variants roGFP1 and roGFP2, however, are particularly suitable only in the reducing compartments of wild-type plants and mutants with limited effects on the amount of glutathione (Meyer et al., 2007; Maughan et al., 2010), or mutants affected in the GSH/GSSG redox equilibrium due to lack of GR (Marty et al., 2009). In more oxidizing compartments or more severe mutants affected in glutathione redox homeostasis, *E*_*GSH*_ is outside the measuring range of these probes thus limiting their usability. The need for less reducing probe variants has been partially fulfilled through the introduction of roGFP1-iX variants derived from roGFP1 (Lohman and Remington, 2008). However, even the least reducing variant roGFP1-iL is still largely oxidized in the ER of human cells and thus hardly capable of detecting oxidative processes in the ER. In addition, the most oxidizing roGFP1-iX variant roGFP1-iL has been reported as being relatively dim compared to the parent roGFP1 (Avezov et al., 2013; Birk et al., 2013). Similar to the continuous extension of the range of differentially colored fluorescent proteins (Shaner et al., 2005) there is also a demand for redox reporter proteins with midpoint potentials matching the *E*_*GSH*_ in distinct compartments or in mutants with targeted alterations in glutathione homeostasis.

The insertion of amino acids in the GFP backbone may lead to changes in the spectroscopic properties of GFP but still yield functional fluorescent proteins (Topell et al., 1999; Cannon and Remington, 2006; Lohman and Remington, 2008). Insertion of single amino acid residues within the roGFP backbone, adjacent to the reactive cysteine 147, leads to a destabilization of the disulfide formed between Cys147 and Cys204. As a consequence, the resulting roGFP1-iX variants show a less negative midpoint potential compared to the parental roGFP1. However, the substitution of the chromophore phenol(ate) interacting His148 by Ser148 was required to maintain ratiometric behavior (Lohman and Remington, 2008). A roGFP1-iL carrying the mutations S147CL and H148S has been reported to have the least negative midpoint potential (−229 ± 5 mV) of a whole series or roGFP1-iX variants (Lohman and Remington, 2008). Introduction of the same changes into roGFP2 lead to roGFP2-iL. This new variant maintained the general ratiometric behavior of roGFP2 with the excitation peak for the anionic form of the chromophore at 488 nm being more pronounced than the UV excitation peak for the protonated chromophore. However, the dynamic range δ for the maximum change in peak excitation ratio was significantly decreased compared to roGFP2 (see Figure 2 and Table 1). A similar reduction of the dynamic range due to amino acid insertion next to Cys147 has already been reported for several roGFP1-iX variants but not for the roGFP1-iL version (Lohman and Remington, 2008). Similarly to roGFP1-iL, roGFP2-iL is also lower in fluorescence than the respective parent roGFP. Nevertheless, the maintenance of ratiometric properties and a sufficiently large dynamic range to resolve biologically relevant redox changes suggest that roGFP2-iL is generally suitable for *in vivo* measurements.

The roGFP1-iL and roGFP2-iL only differ in the S65T substitution introducing an additional methyl group in roGFP2. This minor change not only affects the protonation of the chromophore leading to pronounced stabilization of the anionic form of the chromophore (Elsliger et al., 1999; Jung et al., 2005) but also shifts the midpoint potential of roGFP2 −10 mV less negative compared to roGFP1. The shift in *E*°′ from −280 mV for roGFP2 to −238 mV for roGFP2-iL is lower than the shift for roGFP1-iL which at −229 mV is 62 mV less negative than its parent roGFP1. Different from the original expectation, the modification of roGFP2 did thus not result in a linear additive effect in which the resulting roGFP2-iL was expected to be slightly more negative than roGFP1-iL. The deviation from linearity is probably due to geometric constraints in the roGFP barrel similar to those described for roGFP1-iR which had been expected to be less reducing than roGFP1-iL due to stabilization of the thiolate anion by the introduced adjacent basic arginine residue (Lohman and Remington, 2008). The roGFP1-iR, however, turned out to be more reducing than roGFP1-iL. Whether introduction of basic amino acids in the vicinity of the redox active cysteines may cause a stabilization of thiolates and thus render the redox potential of roGFP2-iL less negative is not known at this stage.

For live cell measurements biosensors should ideally exhibit a strong preference for one specific analyte. By definition, roGFPs undergo thiol/disulfide exchange reactions and it has been shown that specificity for glutathione is achieved through specific interaction with GRXs (Meyer et al., 2007). Fusion of GRX1 to roGFP2 leads to a permanent increase of the local GRX concentration around roGFP and hence this kinetic coupling further increases the likelihood of interaction between roGFP and the fused GRX1 compared to other non-specific interactions with other oxidoreductases (Gutscher et al., 2008; Albrecht et al., 2013). Human GRX1 does also interact with roGFP2-iL and the fusion protein GRX1-roGFP2-iL responds much faster to changes in *E*_*GSH*_ than free roGFP2-iL. Interestingly, fusion of GRX1 to the N-termini of different roGFP variants consistently leads to a slight shift of 5–13 mV in the midpoint potentials toward more negative values. A similar shift of 6 mV has also been reported for GRX1-roGFP1-iE (Birk et al., 2013). Consistent with the respective order of redox potentials, Birk et al. (2013) also observed a higher degree of oxidation of GRX1-roGFP1-iE than for free roGFP1-iE expressed in the ER. A possible explanation for the effects of N-terminally fused GRX on the *E*°′ of roGFP probes might be a mechanical strain that acts on the GFP barrel slightly affecting the thermodynamic stability of the disulfide. Despite the slightly less negative midpoint potential of roGFP1-iL compared with roGFP2-iL, the kinetic properties of both probes for reduction by GSH are very similar. This suggests that both probes may be equally suitable in compartments with oxidizing conditions. RoGFP2-iL thus extends the range of suitable probes for oxidizing compartments by offering additional spectral features. The dominant excitation peak at 488 nm may be of particular advantage for lifetime imaging of roGFPs with pulsed blue excitation. This imaging approach has been successfully used for the original roGFP2 probe and different roGFP1-iX variants (Wierer et al., 2012; Avezov et al., 2013). For roGFP2-iL, excitation with blue light would maximize the excitation and hence minimize the integration time required to sample sufficient photons for analysis. Furthermore, it has been reported that roGFP1 under conditions of intense illumination undergoes an irreversible photoswitch reaction that would foster the anionic form of the chromophore and thus may artificially report reducing conditions even though the local environment of the probe is oxidizing (Schwarzländer et al., 2008).

Generally, roGFP probes are appropriate for measuring the thiol/disulfide equilibrium within a linear range of about ±30 mV from their midpoint potential equivalent to an *OxD* between 10 and 90%. RoGFP2-iL may thus be suitable in the range from ~ −205 to ~ −275 mV. Recently it was shown that GRX1-roGFP1-iE is still oxidized to more than 90% when expressed in the ER of HeLa cells (Birk et al., 2013). This high degree of oxidation clearly limits the use of the probe in that it would not allow investigating processes leading to increasing oxidation in the ER. Based on the even slightly more negative midpoint potential a very similar response can be expected for GRX1-roGFP2-iL.

Stable expression of GRX1-roGFP2-iL and GRX1-roGFP2 in the cytosol of homozygous *rml1* plants allowed ratiometric measurements of the yet undefined *E*_*GSH*_ with both sensors. In both cases the measured values are close to the end of the linear range of the respective probes with GRX1-roGFP2 being even slightly beyond its useful linear measurement range with an oxidation of 93%. GRX1-roGFP2-iL with *OxD* = 14% is still in the linear range. Importantly, *OxD* for both sensor variants can be converted to the respective redox potential and in both cases the redox potential is about −260 mV. A combination of both probes thus effectively doubles the useful dynamic range without leaving a gap between the two probes. The concentration of cytosolic glutathione in wild-type Arabidopsis root tips has been shown to be between 2 and 3 mM (Fricker et al., 2000). Assuming a medium concentration of 2.5 mM cytosolic GSH and an *OxD*_*GSH*_ of 0.002% (Meyer et al., 2007) the Nernst equation predicts an *E*_*GSH*_ of about −310 mV. A remaining GSH level of only 2% in *rml1* would then lead to an *E*_*GSH*_ of −260 mV. This calculation assumes a constant *OxD*_*GSH*_ which is not necessarily given. Obviously, the *rml1* mutant seedlings are stressed and supply with redox equivalents in form of NADPH may be restricted under these severe metabolic constraints. If indeed *OxD*_*GSH*_ was higher in *rml1* than in the wild-type then the total glutathione level in the cytosol would have to be assumed to be slightly above 2% in order to reach an *E*_*GSH*_ of −260 mV. This, however, is still well within the measured range of glutathione concentrations in *rml1*.

In conclusion, GRX1-roGFP2-iL further extends the range of roGFP-based probes for measurements of *E*_*GSH*_ far less reducing than typical *E*_*GSH*_ values normally found in the cytosol. This applies similarly to cell-type specific developmental differences as well as to mutants with low glutathione levels and to generally oxidizing compartments like the ER. GRX1-roGFP2-iL may still be largely oxidized in the ER of non-stressed plants as it has been shown for GRX1-roGFP1-iE in HeLa cells (Birk et al., 2013). The oxidizing redox potential in the lumen supports protein folding whereas deviations from steady-state redox conditions induce an unfolded protein response (Merksamer et al., 2008). Vice versa, malfunction in oxidative protein folding is assumed to affect the redox potential of the lumenal glutathione pool. Under stress situations and in mutants causing a shift in the ER redox potential toward more negative values, GRX1-roGFP2-iL will likely allow dynamic measurement of the glutathione-related redox processes in the ER lumen. Conversely, severe stress situations can cause very strong oxidation in the cytosol, chloroplasts, mitochondria, and peroxisomes and the extent of these reactions may also be dependent on the developmental state of particular cells. Dark-induced senescence has been shown to cause an oxidation of mitochondrial roGFP1 and roGFP2 up to the end of their linear range within only 3 days (Rosenwasser et al., 2010). Under such pathological conditions GRX1-roGFP2-iL will allow to further investigate the most oxidizing phases of the responses.

### Conflict of interest statement

The authors declare that the research was conducted in the absence of any commercial or financial relationships that could be construed as a potential conflict of interest.
